# Worldwide Vaccination Willingness for COVID-19: A Systematic Review and Meta-Analysis

**DOI:** 10.3390/vaccines9101071

**Published:** 2021-09-24

**Authors:** Kimberly R. Nehal, Lieke M. Steendam, Maiza Campos Ponce, Marinka van der Hoeven, G. Suzanne A. Smit

**Affiliations:** Department of Health Sciences, Infectious Disease and Public Health, Vrije Universiteit, 1081 HV Amsterdam, The Netherlands; k.r.nehal@vu.nl (K.R.N.); l.m.steendam@student.vu.nl (L.M.S.); m.vander.hoeven@vu.nl (M.v.d.H.); g.s.a.smit@vu.nl (G.S.A.S.)

**Keywords:** COVID-19, SARS-CoV-2, vaccine, vaccination willingness, vaccination hesitancy, determinants

## Abstract

Countries across the globe are currently experiencing a third or fourth wave of SARS-CoV-2 infections; therefore, the need for effective vaccination campaigns is higher than ever. However, effectiveness of these campaigns in disease reduction is highly dependent on vaccination uptake and coverage in susceptible populations. Therefore, this systematic review and meta-analysis estimated the vaccination intention and identified determinants of willingness and hesitancy. This study updates the existing body of literature on vaccination willingness, and was conducted according to the PRISMA guidelines. PubMed was searched for publications, selecting only studies published between 20 October 2020 and 1 March 2021, in English, with participants aged >16 years of age. The search identified 411 articles, of which 63 surveys were included that accounted for more than 30 countries worldwide. The global COVID-19 vaccination willingness was estimated at 66.01% [95% CI: 60.76–70.89% I^2^ = 99.4% [99.3%; 99.4%]; *τ*^2^ = 0.83]. The vaccination willingness varied within as well as between countries. Age, gender, education, attitudes and perceptions about vaccines were most frequently observed to be significantly associated with vaccine acceptance or refusal.

## 1. Introduction

Vaccine hesitancy is defined as the “delay in acceptance or refusal of vaccination despite availability of vaccination services” [1]. Rises in cases of vaccine-preventable diseases have been related to declines in vaccine coverage. A recent narrative review showed that the majority of measles outbreaks in the United States and Canada were associated with unvaccinated individuals [2,3]. In 2019, the World Health Organization (WHO) declared vaccine hesitancy as amongst the top ten threats to global health [4]. A previous systematic review studying the global perspectives on hesitancy from 2014 found that determinants of, and factors relating to, hesitancy are highly context-specific; varying across place, time, as well as the type of vaccine [5]. 

Meanwhile, the contemporary COVID-19 pandemic has emphasized the importance of a global effort to obtain disease control, in which vaccination plays a key role. Vaccination uptake, however, is highly dependent on vaccine acceptance, and is ultimately instrumental in achieving herd immunity: a critical threshold for disease control. COVID-19 vaccination campaigns have been initiated since December 2020 [6]; however, concerns about vaccination hesitancy had been raised even prior to the start of these campaigns. In multiple surveys, individuals expressed their worries about the safety of the vaccine, due to its rapid development [7]. Other studies reported that individuals deemed the vaccine unnecessary, because they believed they were already immune [8]. 

Even before the approval and roll-out of COVID-19 vaccines, multiple studies have investigated vaccination willingness and/or hesitancy within several populations. In their editorial based on a multi-country European study, Neumann-Böhme et al. [9] reported that vaccination willingness ranged from 62% to 80%. Not only did willingness vary between countries, it also differed between males and females, and different age groups [9]. Other studies in Hong Kong, Israel and the United States reported that vaccination intention was associated with education, political views and previous (influenza) vaccine uptake [10,11,12].

Lin, Tu and Beitsch [13] studied trends in vaccination willingness in over 120 articles up to October 2020. They reported a declining vaccination acceptance, from >70% in March 2020 to <50% in October 2020. Additionally, Wang et al. found an acceptance rate of 73% in a systematic review and meta-analysis, but also showed that the rates differed within their study period of February to September 2020 [14]. This change in acceptance rate over time emphasizes the need to update the existing body of evidence, gaining insight in the current status of vaccine acceptance, globally. 

Therefore, the aim of this study was to (1) estimate the global vaccination willingness by means of a systematic review and meta-analysis, and (2) gain insight in the determinants of vaccination willingness and/or hesitancy. Assessment of vaccination willingness may serve as an indicator of vaccination uptake. Consequently, insights into vaccination-hesitant populations and their characteristics could aid in identifying gaps. This is potentially a first step in tailoring vaccination campaigns in the efforts to increase vaccination awareness, and ultimately, vaccination coverage. 

## 2. Materials and Methods

This systematic review and meta-analysis aimed to estimate the worldwide vaccination willingness against SARS-CoV-2. We also studied the selected literature to assess the determinants that were associated with vaccine hesitancy and acceptance. The study was conducted according to the Preferred Reporting Items for Systematic Reviews and Meta-Analyses (PRISMA) guidelines. 

### 2.1. Search Strategy

PubMed [https://pubmed.ncbi.nlm.nih.gov/ accessed on 20 September 2021] was searched on 16 March 2021 for articles published between 20 October 2020 and 1 March 2021. Table 1 describes the search string used. To ensure comparability, the search string was adapted based on the search strategy of Lin, Tu and Beitsch [13]. The search made use of indexing (MeSH) terms and free text searches and was based on three topics and their synonyms: SARS-CoV-2, vaccination, and survey. To facilitate the selection process, the results of the search (title, abstract and reference of the articles) were uploaded to Microsoft Excel (v. 2104).

### 2.2. Eligibility Criteria

Primary articles were selected if published in English, peer-reviewed and when the full text was available. Only studies with adult participants (>16 years of age) and at least one question on either COVID-19 vaccine willingness or hesitancy were included. Studies with subgroup-specific samples (e.g., nurses, patients, or parents) were excluded from selection. Similarly, reviews, meta-analyses, editorials, as well as other articles involving personal opinions or author’s views were omitted. There were no restrictions based on region or country of the study population. Titles and abstracts were scanned for eligibility. Finally, to determine eligibility, the full text was read. 

### 2.3. Data Extraction

Data extraction was performed sequentially by two authors, L.M.S. and K.R.N.; any discrepancies were discussed with G.S.A.S. until consensus was reached. The process involved retrieving article information including the reference, title, first author, and publication date; demographic data including the country, region, gender, and age; survey characteristics including the survey type, method of contact, survey collection dates, main survey question, and answer options; and the results including vaccination willingness and vaccination hesitancy rates, sample size, and reported bias. When a study consisted of more than one survey with different study populations, all results were extracted. For studies with more than one survey conducted on the same study population, only the most recent survey results were included. 

Vaccination willingness was defined as the proportion of participants willing or intending to receive a vaccine (if or when available). Responses indicating a positive tendency towards vaccinate acceptance, or a negative tendency on a question regarding vaccine refusal or hesitancy, were considered as vaccination-willing (see full overview of categorization of response options in Table 2). 

To gain insight into the determinants of vaccination willingness, determinant analyses were performed for all selected articles. Initially, original questionnaires were checked to retrieve information on potential determinants. If survey questionnaires were unavailable, the full text article was read to retrieve information on measurements and determinants. Measurements were categorized as: significantly associated with the outcome (vaccine acceptance or refusal), not significantly associated with the outcome, unclear if a relationship with the outcome was investigated, or a relationship with the outcome was not investigated. Determinants were included when the association was investigated with a multivariable regression model. Only if the article did not present results of a multivariable regression, were estimates of univariable analyses considered. 

### 2.4. Quality Assessment

To critically appraise the methodological quality of every study included in the systematic review and meta-analysis, the Joanna Briggs Institute (JBI) Critical Appraisal Tool Checklist for Prevalence Studies was used [15]. This checklist was deemed to fit the aim of the systematic review best. The checklist consisted of nine questions on the methods (e.g., sample selection, sample size, valid and reliable measurements) and applied analyses of a study. The appraisal tool aids in the determination of what articles should be either included or excluded, or whether more information should be sought [16]. 

### 2.5. Data Analysis

To estimate global vaccination willingness, a random-effect meta-analysis of single proportions was performed. Similar meta-analyses were performed to estimate vaccination willingness per, continent, if multiple surveys were conducted. 

A random intercept logistic regression model (or generalized linear mixed model, GLMM) was used for the meta-analysis, after logit transformation of the data. The within-study variation was estimated with the Clopper–Pearson 95% confidence interval (CI) and the between-study variation was estimated with the maximum likelihood estimator for tau^2^. The Higgin’s and Thompsons’s I^2^ was used to assess heterogeneity. Statistical analyses were performed, and the forest and funnel plots were created using R Statistical Software version 4.0.5. [17]. Microsoft Excel was used for determinant assessment. 

## 3. Results

### 3.1. Search Results

**Figure 1:**
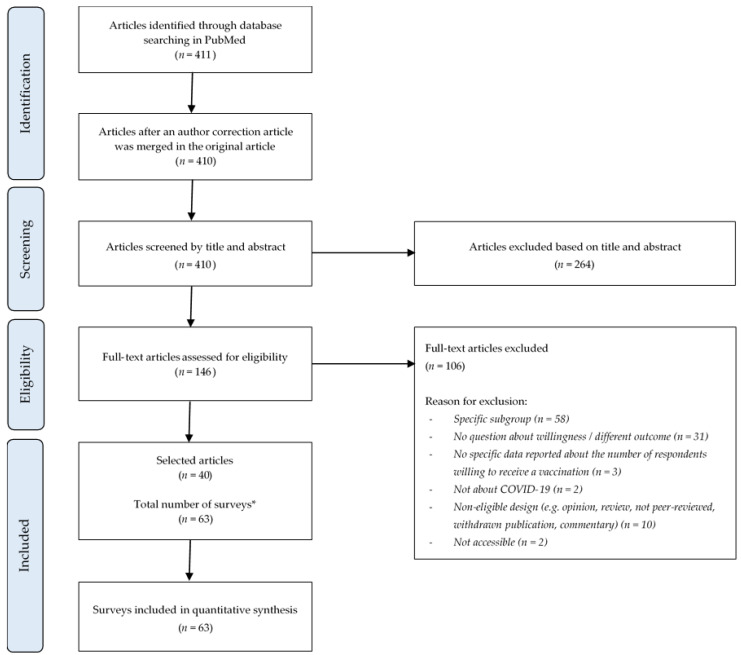
PRISMA flow diagram of study search and selection process. * Some articles described the results of multiple surveys.

The data search identified 411 articles. One article was found to be an author’s correction on another article, and was therefore merged with the original [18]. After assessing eligibility based on the title and abstract or the full text, 40 articles were included in the final selection. Amongst these, 4 articles were found to contain results of multiple surveys. Lazarus et al. conducted a survey in 19 countries worldwide [18]. Sallam et al. questioned participants in Jordan, Kuwait, Saudi Arabia and other Arabic-speaking countries [19]. Murphy et al. conducted a survey in Ireland and the United Kingdom [20]. Lastly, the study of Wang et al. performed two surveys with different samples at two different time points [21]. This meant that the results of a total of 63 surveys were included in the meta-analysis (Figure 1). 

### 3.2. Study Characteristics 

The vast majority of studies had a cross-sectional design, where data and measurements were retrieved via a telephone or online survey. Some studies recruited participants via existing databases, and others conducted snowball sampling (through social media, or email) as the main recruitment method. Surveys were performed between March 2020 and December 2020, with the most surveys performed in June (*n* = 10) [18,22,23,24,25,26,27,28,29,30]. One study was conducted after the initiation of vaccination campaigns. However, campaigns had not rolled out; therefore, vaccines were not yet available in the countries of the surveyed participants [19]. The sample size varied from 113 participants [31] to 5114 participants per survey [32]. In two studies, participants were asked a question on vaccine hesitancy/refusal, instead of acceptance [20,33]. 

Populations in the United States (USA) were most frequently studied, with eight studies recruiting their participants in the United States [18,25,29,30,31,34,35,36]. This was followed by Italy [18,24,37,38,39]; Italian samples were studied in five articles. An overview of the included studies and study characteristics is presented in Table 3.

### 3.3. Worldwide Vaccination Willingness

**Figure 2:**
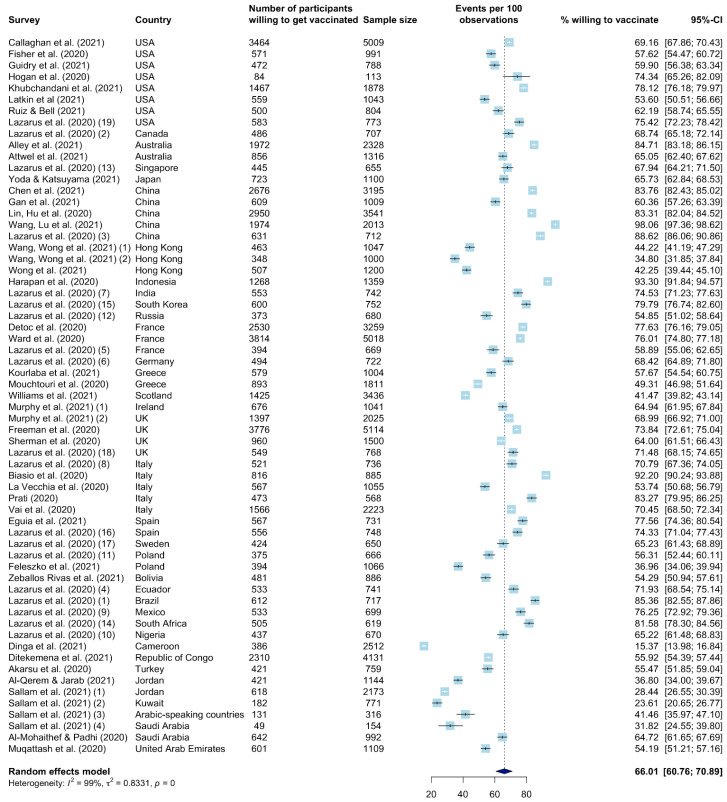
Forest plot of the worldwide vaccination willingness rate.

The estimated worldwide COVID-19 vaccination willingness was 66.01% (95% CI: 60.76–70.89% I^2^ = 99.4% (99.3%; 99.4%); *τ*^2^ = 0.83) (Figure 2). The highest vaccination willingness of 98.06% (95% CI: 97.36–98.62%) was observed in the study of Wang, Lu et al. [53]. The Cameroon study of Dinga et al. reported the lowest vaccination willingness: 15.37% (95% CI: 13.98–16.84%) [27]. 

The continent-specific vaccination willingness in Asia, Europe, and North America were close to the global willingness (Supplementary Material, Figure S1.1–1.6). Out of the 63 surveys, 22 were conducted in populations on the Asian continent (including the Middle East region). The vaccination willingness for these 22 surveys was estimated at 63.71% (95% CI: 51.25–74.57%). A total of 22 surveys were also included for Europe, where the vaccination willingness was estimated at 67.42% (95% CI: 61.32–72.98%). Based on nine surveys, the vaccination willingness in North America was estimated at 66.89% (95% CI: 61.26–72.09%). 

The highest willingness was observed in South America, 73.27% (95% CI: 61.03–82.75%), based on four surveys, and Oceania, 76.27% (95% CI: 60.13–87.25%), based on two surveys (in Australia). Based on four surveys, the lowest willingnesswas estimated for (sub-Saharan) Africa: 54.02% (95% CI: 27.16–78.72%). I^2^ values ranged from 97.2% (95% CI: 96.1–98.0%) for North America, to 99.8% (95% CI: 99.7–99.8%) in Africa. 

### 3.4. Determinant Assessment

The second aim of the review was to gain insight into determinants of vaccination willingness and/or hesitancy. An overview of the most frequently studied determinants is presented in Table 4. Out of the 40 included articles, 12 did not perform univariable or multivariable regression analyses with vaccination willingness as outcome. Therefore, these were not included in Table 4. Although only two articles questioned vaccine hesitancy/refusal rather than acceptance, multiple papers changed the reference category to study determinants of hesitancy/refusal or lower vaccine acceptance. We observed that age, gender, education and attitudes around vaccines were most frequently significantly associated with either vaccine acceptance or refusal amongst the surveys that studied these determinants.

All 28 papers studied gender and age. In 16 out of 28, a significant association was found between gender and vaccine acceptance or hesitancy. Age was found to be significantly associated with vaccine acceptance or hesitancy in 13 papers, and was borderline significant in 1 study [42]. Associations were found for ethnicity (in some articles also specified as race), but the majority (10 out of 12) of these articles were on populations in the United States or the United Kingdom. 

Education was studied in 25 articles, of which 14 found a (borderline) significant association. All 10 papers that reported on the benefits, harms and/or barriers of vaccines and/or other beliefs and attitudes about vaccines reported significant associations with vaccination willingness or hesitancy. Out of the 9 papers that studied partisan or political preferences, 8 studies found a significant association with acceptance or hesitancy. Although 14 articles investigated living location (either urbanity or geography) in a regression analysis, only 5 found a (borderline) significant association. 

### 3.5. Risk of Bias

The methodological quality and risk of bias was checked for all 40 included articles using the JBI checklist for prevalence studies. A large proportion of studies used suboptimal recruitment methods (e.g., convenience and snowball sampling via social media), which may have led to a non-representative sample of the national population, although no studies were excluded. 

To assess publication bias, a funnel plot was made (Supplementary Material, Figure S2). Out of the 63 included surveys, 42 were found to be outliers compared to the mean estimated global vaccination willingness. 

## 4. Discussion

This systematic review and meta-analysis of 63 surveys estimated a global vaccination willingness of 66.01% [95% CI: 60.76–70.89%]. Additionally, several determinants were significantly associated with vaccine acceptance and hesitancy. Age, gender, education as well as attitudes and beliefs about vaccination were observed to be most frequently related to the respondent’s intention to receive or refuse a vaccine when available.

However, differences in acceptance rates between and within continents and countries were observed. This estimated global vaccination willingness of 66.01% is lower than the estimates presented by Wang et al. [14]. Based on their systematic review including studies up to November 4, 2020, they reported a pooled vaccine acceptance rate of 73.31% [95% CI: 70.52–76.01%]. Multiple studies have suggested that vaccine acceptance changes over time. Sallam et al. reported vaccine acceptance to vary from 56.9% in April 2020 to 75.4% in June in the United States [58]. The multi-country survey of Neumann-Böhme et al. [9] reported a 20% decline in acceptance across multiple European countries during their study period.

Similar to the study of Wang et al. [14], the observed variation in our review across studies due to heterogeneity was high. Wang et al. found an I^2^ value of 98.8% (no 95% CI presented), compared to 99.4% in our study. To account for heterogeneity, a random-effects model was used. Differences in time, place, and between populations were expected, but could not be explained due to clinical differences, for example, nor could they be investigated using subgroup analyses [59]. The heterogeneity observed in our study could not be eliminated in the continental analyses, with I^2^ values for all stratified analyses exceeding 95%.

In the determinant assessment, we observed significant associations with gender and vaccination hesitance or acceptance in multiple studies. Prior to the COVID-19 pandemic, a review reported that females were less likely to accept vaccination compared to males for tetanus, diphtheria, and pertussis (Td(ap)) and influenza [60]. This has been confirmed for COVID-19 [21,23,25,26,29,30,32,34,36,41,42,61,62,63]. Unfounded rumors about vaccines having detrimental effects on fertility could explain why vaccination uptake and intention is lower in women. During the pandemic, similar concerns have been raised by (young) women as a reason for hesitancy [64].

Attitudes and perceptions, including perceptions on the benefits, harms and barriers of vaccines, were significantly associated with acceptance and hesitance. In line with the expectation, those with positive attitudes and perceptions of the COVID-19 vaccine or vaccines in general were more likely to accept and less likely to refuse, based on the findings of our review [14,19,25,26,35,43,49,52,55], as well as in other studies [61,65,66]. More specifically, and related to the rumors on infertility, concerns about the efficacy and possible side-effects seem to play a major role in the intention to vaccinate [61,65,66].

Healthcare system distrust as well as vaccine hesitancy are suggested to be closely related to health literacy [67]. Heidari et al. [68] emphasized the need for an intersectional gender approach to vaccine development and deployment. They recognize health literacy as one of the issues creating gender-related barriers to vaccination. Additionally, access to healthcare may influence vaccination willingness. The study of Bianconi et al. has discussed the potential role of gender inequalities in the access to healthcare facilities in COVID-19 testing and diagnostics [69]. Investigating similar barriers and inequities in healthcare (facility) access could be of interest during vaccine deployment [68].

To the best of our knowledge, this is the most recent systematic review and meta-analysis on vaccination willingness and hesitancy including related determinants. Previous studies have included papers published up to November 2020.

Our systematic review and meta-analysis is, however, subject to some limitations. To obtain the largest possible number of studies, we included studies with many different types of sampling methods. The majority of included studies used convenience sampling when recruiting participants. It is unclear how sampling methods may have influenced vaccination willingness, and whether these results may have been different if sampling would have been more purposeful. Concerns could be raised about minority and underrepresented groups that may not be reached through convenience and snowball sampling methods [70].

In addition, although we did not apply geographical restrictions during the inclusion of articles, the final selection did show an overrepresentation of (Eastern) Asian and European studies. A study in nine low- and middle-income countries (LMICs) showed acceptance ranging between 23% (Benin) and 89% (Brazil) when the available vaccine was 90% effective and ranging between 48% (Benin) and 94% (Brazil) with a vaccine effectivity of 95% [71]. In their 2012 editorial, Thomson and Watson stated that vaccine adoption = access + acceptance [72].

Finally, in our determinant assessment, we could not provide full insights into the direction of associations. This is in line with earlier research which showed that determinants of vaccine hesitancy are highly context-specific [5]. This, together with high heterogeneity found in our study, as well as in the review by Wang et al. [14], imply substantial differences between (sub)populations. Therefore, strategies focusing on removing vaccination barriers should follow an approach that is in line with determinants that are relevant within their specific setting and population.

Future studies should therefore focus on the determinants observed in this study (age, gender, education as well as attitudes and beliefs about vaccination), yet with particular attention to context-specific conditions. This may reveal insights into (sub)population-specific vaccination hesitancy and thus help vaccination programmes to increase vaccination coverage.

## Figures and Tables

**Table 1:** PubMed search string, adapted from Lin, Tu and Beitsch [13] (continued on the next page).

(“coronavirus”[MeSH Terms] OR “coronavirus”[All Fields] OR “coronaviruses”[All Fields] OR “COVID 19”[All Fields] OR “SARS-2”[All Fields] OR “severe acute respiratory syndrome coronavirus 2”[All Fields] OR “severe acute respiratory syndrome coronavirus 2”[Supplementary Concept] OR “ncov”[All Fields] OR “2019 ncov”[All Fields] OR “sars cov 2”[All Fields]) AND (“vaccines”[MeSH Terms] OR “vaccin”[All Fields] OR “vaccination”[MeSH Terms] OR “vaccination”[All Fields] OR “vaccinable”[All Fields] OR “vaccinal”[All Fields] OR “vaccinate”[All Fields] OR “vaccinated”[All Fields] OR “vaccinates”[All Fields] OR “vaccinating”[All Fields] OR “vaccinations”[All Fields] OR “vaccination’s”[All Fields] OR “vaccinator”[All Fields] OR “vaccinators”[All Fields] OR “vaccine s”[All Fields] OR “vaccined”[All Fields] OR “vaccines”[All Fields] OR “vaccine”[All Fields] OR “vaccins”[All Fields] OR “vaccin”[Supplementary Concept]) AND (“surveys and questionnaires”[MeSH Terms] OR “survey”[All Fields] OR “surveys”[All Fields] OR “survey’s”[All Fields] OR “surveyed”[All Fields] OR “surveying”[All Fields] OR (“surveys”[All Fields] AND “questionnaires”[All Fields]) OR “surveys and questionnaires”[All Fields] OR (“questionnair”[All Fields] OR “questionnaire’s”[All Fields] OR “surveys and questionnaires”[MeSH Terms] OR (“surveys”[All Fields] AND “questionnaires”[All Fields]) OR “surveys and questionnaires”[All Fields] OR “questionnaire”[All Fields] OR “questionnaires”[All Fields]) OR “poll”[All Fields])

**Table 2:** Survey question responses categorized as vaccination-willing.

Questioned	Responses
Vaccination willingness	yes/yes, I will be vaccinated/yes if it’s free/strongly agree/agree/yes certainly/yes possibly/definitely/probably/definitely planning/probably planning/yes (only when the vaccine is provided for free)/yes (willing to pay for a vaccine)/very likely/somewhat likely/probably yes/completely agree/somewhat agree/definitely yes/quite a bit/extremely likely/8–10 (very likely)/I probably would want to receive it/I definitely would want to receive it/high 6–7
Vaccination refusal or hesitancy	probably not/certainly not/accepting of a COVID-19 vaccine

**Table 3:** Study characteristics of included articles in the systematic review, meta-analysis and determinant analysis.

Author	Country	Survey Date	Main Survey Question	Answer Options	Vaccination Willing (*n*)	Sample Size (*n*)
Akarsu et al. (2020) [21]	Turkey	10 June 2020–20 July 2020	If a vaccine for COVID-19 is developed, would you be vaccinated against COVID-19?	yes, I will be vaccinated/yes, if it’s free/no, I don’t/undecided	421	759
Alley et al. (2021) [23]	Australia	9 April 2020–16 August 2020	If a new vaccine for COVID-19 was released that was proven to be safe and effective, I would get vaccinated immediately.	strongly agree/agree/neither agree nor disagree/disagree/strongly disagree	1972	2328
Al-Mohaithef and Padhi (2020) [40]	Saudi Arabia	Not reported	If a vaccine against coronavirus is available, I will take it.	yes/no/not sure	642	992
Al-Qerem and Jarab (2021) [41]	Jordan	1 October 2020–31 October 2020	If a vaccine is available for COVID-19, are you willing to take it?	yes/no/not sure	421	1144
Attwel et al. (2021) [42]	Australia	18 May 2020–29 May 2020	If a COVID-19 vaccine were available today, would you get it?	yes/maybe/no	856 *	1316
Biasio et al. (2020) [24]	Italy	5 June 2020–13 June 2020	Will you get vaccinated, if possible?	yes/no	816	885
Callaghan et al. (2021) [25]	USA	28 May 2020–08 June 2020	If a COVID-19 vaccine is developed, would you pursue getting vaccinated for the coronavirus?	yes/no	3464 *^/^**	5009
Chen et al. (2021) [26]	China	1 May 2020–30 June 2020	Willing to receive a COVID-19 vaccine.	yes/no/unsure	2676	3195
Detoc et al. (2020) [43]	France	26 March 2020–20 April 2020	If a vaccine against the new coronavirus was available for next season, would you get vaccinated?	yes, certainly/yes, possibly/I don’t know/no, possibly/definitely no	2530	3259
Dinga et al. (2021) [27]	Cameroon	1 May 2020–31 August 2020	Would you agree to receive a COVID-19 vaccine?	yes/no/I will need more information/I don’t know	386	2512
Ditekemena et al. (2021) [44]	Republic of Congo	24 August 2020–8 September 2020	Would you consent to receive a COVID-19 vaccine if it becomes available in our country?	yes/no	2310	4131
Eguia et al. (2021) [45]	Spain	10 September 2020–23 November 2020	Intention to get vaccinated.	yes/no	567	731
Feleszko et al. (2021) [28]	Poland	2 June 2020–9 June 2020	If a vaccine against coronavirus disease 2019 (COVID-19) is available and safe, do you plan to vaccinate?	yes/no/I do not know or it is difficult to answer	394	1066
Fisher et al. (2020) [34]	USA	16 April 2020–20 April 2020	When a vaccine for the coronavirus becomes available, will you get vaccinated?	yes/no/not sure	571	991
Freeman et al. (2020) [32]	UK	24 September 2020–17 October 2020	Would you take a COVID-19 vaccine (approved for use in the UK) if offered?	definitely/probably/I may or I may not/probably not/definitely not/don’t know	3776	5114
Gan et al. (2021) [46]	China	23 October 2020–10 November 2020	Would you be vaccinated against COVID-19?	yes/no/unsure	609	1009
Guidry et al. (2021) [35]	USA	1 July 2020–31 July 2020	I intend to get the COVID-19 vaccine when it becomes available.	definitely planning/probably planning/neutral/probably not planning/definitely not planning	472 *	788
Harapan et al. (2020) [47]	Indonesia	25 March 2020–6 April 2020	Would you accept a COVID-19 vaccine?	no/yes (only when the vaccine is provided for free)/yes (willing to pay for a vaccine)	1268	1359
Hogan et al. (2020) [31]	USA	1 April 2020–30 April 2020	If a vaccine becomes available for COVID-19, would you get it?	yes/no	84 *	113
Khubchandani et al. (2021) [29]	USA	1 June 2020–30 June 2020	If a vaccine was available that would prevent coronavirus infection, how likely is it that you would get the vaccine/shot?	very likely/somewhat likely/not likely/definitely not	1467	1878
Kourlaba et al. (2021) [48]	Greece	28 April 2020–3 May 2020	Willingness to be vaccinated against Coronavirus if a vaccine was to become available.	yes/no/do not know	579	1004
La Vecchia et al. (2020) [37]	Italy	16 September 2020–28 September 2020	Attitude towards a potential COVID-19 vaccine.	yes/probably yes/probably no/no	567 *	1055
Latkin et al. (2021) [36]	USA	14 May 2020–18 May 2020	If a vaccine against the coronavirus becomes available, do you plan to get vaccinated, or not?	yes, I will get a coronavirus vaccine/no, I will not get a coronavirus vaccine/not sure	559	1043
Lazarus et al. (2020) [18]	Brazil	1 June 2020–30 June 2020 ^•^	Would you take a proven, safe and effective COVID-19 vaccine? ^•^	completely agree/somewhat agree/neutral or no opinion/somewhat disagree/completely disagree ^•^	612	717
	Canada				486	707
	China				631	712
	Ecuador				533	741
	France				394	669
	Germany				494	722
	India				553	742
	Italy				521	736
	Mexico				533	699
	Nigeria				437	670
	Poland				375	666
	Russia				373	680
	Singapore				445	655
	South Africa				505	619
	South Korea				600	752
	Spain				556	748
	Sweden				424	650
	UK				549	768
	USA				583	773
Lin, Hu et al. (2020) [49]	China	1 May 2020–19 May 2020	Intention to take the COVID-19 vaccine.	definitely yes/probably yes/probably no/definitely no	2950	3541
Mouchtouri et al. (2020) [50]	Greece	15 April 2020–02 May 2020	Should a vaccine be available for COVID-19, I will receive it.	definitely yes/yes/maybe/no	893	1811
Muqattash et al. (2020) [51]	United Arab Emirates	4 July 2020–04 August 2020	How willing are you to get the COVID-19 vaccine, once discovered?	not at all/a little/a moderate amount/quite a bit	601	1109
Murphy et al. (2021) [20]	Ireland	Not reported	Prevalence of vaccine hesitancy and resistance in Ireland.	accepting of a COVID-19 vaccine/hesitant about such a vaccine/resistant towards a vaccine	676 *	1041
	UK				1397 *	2025
Prati (2020) [39]	Italy	1 April 2020–30 April 2020	Assume that your local health authority makes a vaccine against SARS-CoV-2 freely available. Do you intend to get the vaccine?	yes/no/do not know	473	568
Ruiz and Bell (2021) [30]	USA	15 June 2020–16 June 2020	All things considered, how likely are you to get a coronavirus vaccine when one becomes available?	extremely likely/somewhat likely/unsure/somewhat unlikely/extreme unlikely	500 *	804
Sallam et al. (2021) [19]	Jordan	14 December 2020–18 December 2020	Will you get the coronavirus vaccine when available?	yes/no	618	2173
	Kuwait				182	771
	Arabic speaking countries ^#^				131	316
	Saudi Arabia				49	154
Sherman et al. (2020) [52]	UK	14 July 2020–17 July 2020	When a coronavirus vaccination becomes available to you, how likely is it you will have one?	0–2 = very unlikely 3–7 = uncertain 8–10 = very likely	960 *	1500
Vai et al. (2020) [38]	Italy	27 February 2020–8 March 2020	Declare if you would have vaccinated for SARS-CoV-2.	yes/no	1566	2223
Wang, Lu et al. (2021) [53]	China	15 November 2020–15 December 2020	Accept vaccination if the COVID-19 vaccine is successfully developed and approved for listing in the future.	yes/no	1974	2013
Wang, Wong et al. (2021) [21]	Hong Kong	17 February 2020–27 February 2020	If a COVID-19 vaccine is available now, whether or not will you choose to accept it.	yes (accept)/no (refuse)/undecided	463 *	1047
		24 August 2020–7 September 2020			348 *	1000
Ward et al. (2020) [33]	France	1 April 2020–30 April 2020	Would you refuse a vaccine against the COVID-19 when available?	certainly/probably/probably not/certainly not	3814	5018
Williams et al. (2021) [54]	Scotland	1 August 2020–31 August 2020	If a vaccine for coronavirus (COVID-19) becomes available, would you want to receive it?	I definitely would not want to receive it/I probably would not want to receive it/unsure/I probably would want to receive it/I definitely would want to receive it	1425	3436
Wong et al. (2021) [55]	Hong Kong	27 July 2020–27 August 2020	If the Government will provide a free-of-charge COVID19 vaccine within the next 12 months, will you receive it?	yes/no/not sure	507	1200
Yoda and Katsuyama (2021) [56]	Japan	1 September 2020–30 September 2020	Would you be willing to be vaccinated when the COVID-19 vaccine is developed?	yes/unsure/no	723	1100
Zeballos Rivas et al. (2021) [57]	Bolivia	29 April 2020–9 May 2020	Would you use a COVID-19 vaccine if it were available?	null or low 1–3/moderate 4–5/high 6–7	481	886

* Number of participants willing to receive COVID-19 vaccination not directly mentioned in the article. The number was calculated by multiplying the proportion of participants willing to receive a vaccine with the total sample size. ** Number of participants willing to receive COVID-19 vaccination not directly mentioned in the article. The number was calculated by subtracting the total number of hesitant participants from the total sample size. ^•^ These questions apply to all studies in Lazarus et al., 2020. ^#^ Other countries with respondents for the survey included (*n*): Palestine = 98, Iraq = 60, United Arab Emirates = 44, Yemen = 30, Qatar = 28, Egypt = 17, Lebanon = 9, Oman = 8, Bahrain = 6, Tunisia = 5, Sudan = 4, Syria = 3, Somalia = 2, Algeria = 1 and Morocco = 1.

**Table 4:** Selected articles (sorted by first author) and studied determinants of vaccine acceptance or hesitancy and/or refusal.

		Alley [23]	Al-Mohaithef [40]	Al-Qerem [41]	Attwell [42]	Callaghan [25] -	Chen [26]	Detoc [43]	Ditekemena [44]	Fisher [34]	Freeman [32]	Gan [46]	Guidry [35]	Khubchandani [29]	Kourlaba [48]	Latkin [36]	Lazarus [18] -	Lin [49]	Murphy [20]	Prati [39]	Ruiz [30]	Sallam [19]	Sherman [52]	Vai [38]	Wang, Wong [21]	Wang, Lu [53]	Ward [33]	Williams [54]	Wong [55]
Sociodemographics	Living location	●	꙱ *	◊			◊ *		◊	▫	● *	●		●	●	●		●/■ *	▫		● *	■ *	◊ *	◊		●			● *
	Ethnicity					▫	■			▫	▫		■	●		▫			▫	●	●		●					■	
	Gender/sex	●	●	▫	▫	▫	■	■	●	▫	▫	●	●	▫	●	▫	■	●	▫	●	■	■	●	●	■	■	▫	●	●
	Age	●	■	●	꙱	●	●	■	◊	▫	▫	■	●	●	●	▫	■	●	▫	▫	●	●	■	▫	■	◊	▫	●	●
	Political preferences					▫					▫		●	▫		▫			▫		■						▫		
	Religion					▫			●		▫		●					●	◊				●						
SES indicators	Education	■	꙱	▫	꙱	●	■		●	▫	▫	■	■	▫	●	●	■				●	■	●	▫	●	◊	●	■	●
	Income/insurance	●		◊	●	▫	■		■	●	▫	●	■	▫		▫	■	●	▫	꙱	■	◊	◊	◊		■	▫	■	●
	Occupation/ Employment status		꙱				●		◊	◊	▫	꙱		▫	■	●		■					●		■	●			●
	Health-related occupation			●			◊	■	■			◊			◊								●						
Family composition	Children in household/household size			●	꙱					●	●			▫	●				◊				◊						
	Marital status		■	▫					◊	◊	꙱				●	●		꙱			●				■	■			
Health status	Risk group or conditions	꙱		●			●	●	◊	●		●			■			■	▫		■	■	●		●	●		■	■
	Vaccine history	◊			▫			◊		▫		■			●						■	◊	■		■	■			
Experiences with COVID-19	Suspected or laboratory COVID-19 diagnosis (self)					▫			■		◊				◊	●	■	●	◊		◊	●	●				●		
	Suspected or laboratory COVID-19 diagnosis (others)						◊		◊						◊				◊				●						
Perceived risk for self or others in direct network	Concerns about (risk of) COVID-19		■	●		▫	●	■	◊				■	▫		●		■		▫	■		●	▫		■	▫		●
	Perceived disease severity			▫	▫		●			▫	▫		●					●					●						■
	(Concerns about) vulnerable household/network								◊					▫	●	●							●						
	Severity of disease in region	■															■									■			
Attitudes, beliefs and perceptions about vaccination	Benefits of vaccines					▫							■					■					●			■			■
	Harm and/or barriers of vaccines						■						■					■					■			■			■
	Other beliefs and attitudes about vaccines					▫	■	■				■	■								■		■						
Attitudes, beliefs and perceptions about COVID-19	COVID-19-related knowledge or behavior			●					◊		◊	●			■	●							■	◊	◊				
	Attitudes and beliefs about COVID-19						◊		◊				■		■							■	■	▫					
	Impact of pandemic on life, work, and/or income								◊		◊	●				▫							●			■			
	Expressions of mistrust about COVID-19 and/or vaccination			◊	▫				■		◊				■				◊	●	■	■							
	Trust in health system/government		■	◊			■										■		◊	▫		◊	●						■
	Trust in science/manufacturer			◊		▫													◊			◊	●						■
Informants	Media use	▫					◊		◊			■			●				◊		■	◊		◊					
	Cues to action (e.g., from others in network, doctors)	◊											■					■					◊			■			■

Symbols indicate if the determinant was: measured, but unclear or not included in a regression analysis (◊); measured but not significantly associated with the outcome of vaccine acceptance/hesitancy (●); borderline significantly associated with the outcome of vaccine acceptance/hesitancy (꙱); and significantly associated with vaccine acceptance (■); significantly associated with vaccine refusal or hesitancy (▫). Papers indicated with (-) have only presented univariable regressions; therefore, the symbols indicate the association found in the univariable regression analyses. * in living location indicates that a geographical location rather than urbanity was measured.

## Data Availability

Data can be requested from the authors via: m.camposponce@vu.nl.

## Supplementary Materials

The following are available online at https://www.mdpi.com/article/10.3390/vaccines9101071/s1, Figure S1.1–S1.6: Forest plots of the continent specific vaccination willingness, Figure S2: Funnel plot of the proportion of the worldwide vaccination willingness.
