# Supplementary material for: Worldwide Vaccination Willingness for COVID-19: A Systematic Review and Meta-Analysis
Source: Vaccines (Basel). 2021 Sep 24;9(10):1071. doi: 10.3390/vaccines9101071 (PMC8540052; doi:10.3390/vaccines9101071)
Supplement: Supplementary file 1 [file vaccines-09-01071-s001.zip › vaccines-1352945-supplementary.pdf]

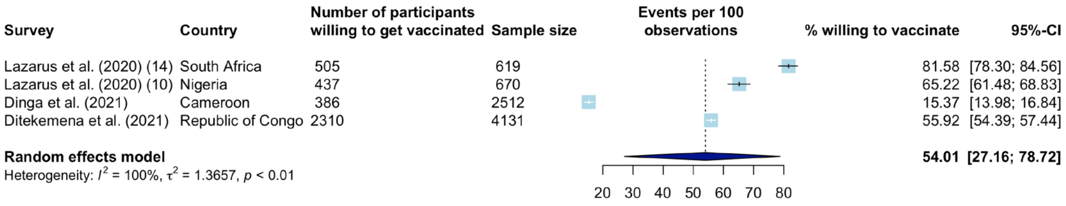

Figure S1.1. Forest plot of vaccination willingness in the African continent

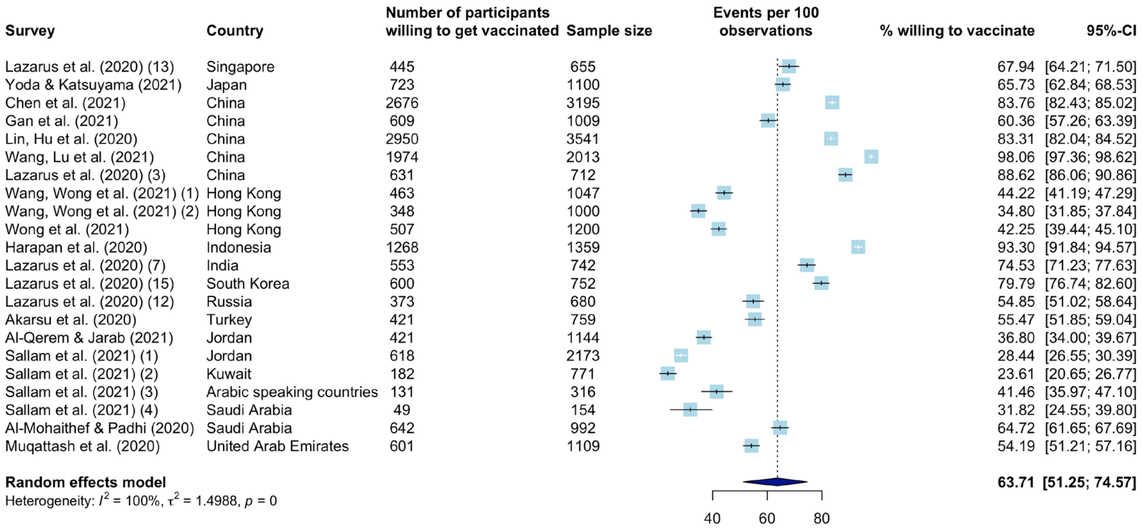

Figure S1.2. Forest plot of vaccination willingness in the Asian continent

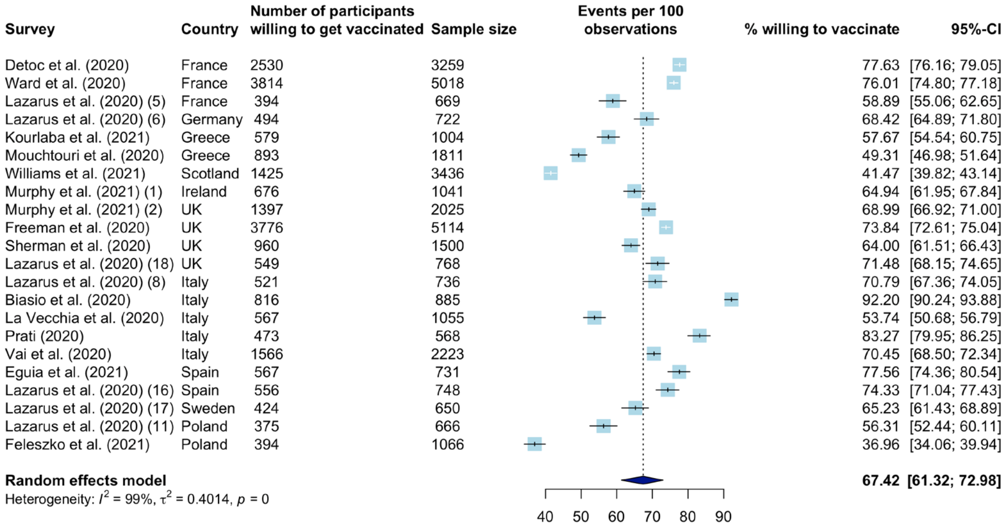

Figure S1.3. Forest plot of vaccination willingness in the European continent

Survey	Country	Number of participants willing to get vaccinated	Sample size	% willing to vaccinate	95%-CI
Callaghan et al. (2021)	USA	3464	5009	69.16	[67.86; 70.43]
Fisher et al. (2020)	USA	571	991	57.62	[54.47; 60.72]
Guidry et al. (2021)	USA	472	788	59.90	[56.38; 63.34]
Hogan et al. (2020)	USA	84	113	74.34	[65.26; 82.09]
Khubchandani et al. (2021)	USA	1467	1878	78.12	[76.18; 79.97]
Latkin et al (2021)	USA	559	1043	53.60	[50.51; 56.66]
Ruiz & Bell (2021)	USA	500	804	62.19	[58.74; 65.55]
Lazarus et al. (2020) (19)	USA	583	773	75.42	[72.23; 78.42]
Lazarus et al. (2020) (2)	Canada	486	707	68.74	[65.18; 72.14]
**Random effects model**				**66.89**	**[61.26; 72.09]**

Heterogeneity: $I^2 = 97\%$, $\tau^2 = 0.1328$, $p < 0.01$

Figure S1.4. Forest plot of vaccination willingness in the North American continent

Survey	Country	Number of participants willing to get vaccinated	Sample size	% willing to vaccinate	95%-CI
Zeballos Rivas et al. (2021)	Bolivia	481	886	54.29	[50.94; 57.61]
Lazarus et al. (2020) (4)	Ecuador	533	741	71.93	[68.54; 75.14]
Lazarus et al. (2020) (1)	Brazil	612	717	85.36	[82.55; 87.86]
Lazarus et al. (2020) (9)	Mexico	533	699	76.25	[72.92; 79.36]
**Random effects model**				**73.27**	**[61.03; 82.75]**

Heterogeneity: $I^2 = 98\%$, $\tau^2 = 0.3186$, $p < 0.01$

Figure S1.5. Forest plot of vaccination willingness in the South American continent

Survey	Country	Number of participants willing to get vaccinated	Sample size	% willing to vaccinate	95%-CI
Alley et al. (2021)	Australia	1972	2328	84.71	[83.18; 86.15]
Attwel et al. (2021)	Australia	856	1316	65.05	[62.40; 67.62]
**Random effects model**				**76.27**	**[60.13; 87.25]**

Heterogeneity: $I^2 = 99\%$, $\tau^2 = 0.2945$, $p < 0.01$

Figure S1.6. Forest plot of vaccination willingness in Oceania

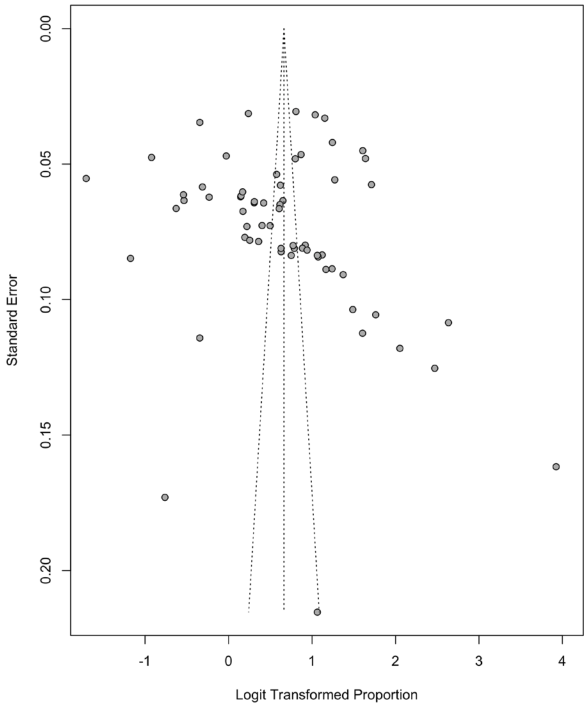

Figure S2. Funnel plot of the proportion of the worldwide vaccination willingness
